# Pericentric major satellite transcription is essential for meiotic chromosome stability and spindle pole organization

**DOI:** 10.1098/rsob.230133

**Published:** 2023-11-08

**Authors:** Claudia Baumann, Xiangyu Zhang, Maria M. Viveiros, Rabindranath De La Fuente

**Affiliations:** ^1^ Department of Physiology and Pharmacology, College of Veterinary Medicine, University of Georgia, Athens, GA 30602-0002, USA; ^2^ Regenerative Biosciences Center (RBC), University of Georgia, Athens, GA 30602-0002, USA

**Keywords:** meiosis, pericentric heterochromatin, major satellite transcripts, non-coding RNA, spindle, aMTOC

## Abstract

In somatic cells, mitotic transcription of major satellite non-coding RNAs is tightly regulated and essential for heterochromatin formation and the maintenance of genome integrity. We recently demonstrated that major satellite transcripts are expressed, and chromatin-bound during mouse oocyte meiosis. Pericentric satellite RNAs are also expressed in human oocytes. However, the specific biological function(s) during oocyte meiosis remain to be established. Here, we use validated locked nucleic acid gapmers for major satellite RNA depletion followed by live cell imaging, and superresolution analysis to determine the role of pericentric non-coding RNAs during female meiosis. Depletion of satellite RNA induces mesoscale changes in pericentric heterochromatin structure leading to chromosome instability, kinetochore attachment errors and abnormal chromosome alignment. Chromosome misalignment is associated with spindle defects, microtubule instability and, unexpectedly, loss of acentriolar microtubule organizing centre (aMTOC) tethering to spindle poles. Pericentrin fragmentation and failure to assemble ring-like aMTOCs with loss of associated polo-like kinase 1 provide critical insight into the mechanisms leading to impaired spindle pole integrity. Inhibition of transcription or RNA splicing phenocopies the chromosome alignment errors and spindle defects, suggesting that pericentric transcription during oocyte meiosis is required to regulate heterochromatin structure, chromosome segregation and maintenance of spindle organization.

## Graphical abstract

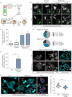

## Highlights

— Pericentric major satellite RNA is transcribed and processed during oocyte maturation.
— Depletion of satellite RNA induces mesoscale changes in heterochromatin organization, defects in microtubule–kinetochore attachments and chromosome alignment errors.
— Loss of major satellite RNA disrupts spindle microtubule stability and spindle pole organization.
— The mechanism(s) involve lack of PLK1 localization at spindle poles.

## 1. Introduction

Pericentric heterochromatin structure and function is critical for genome stability and nuclear architecture in somatic and germ cells [1–5]. Heterochromatin formation is epigenetically regulated and involves repressive histone modifications, chromatin remodelling proteins as well as centromeric non-coding RNAs [6–9]. In somatic cells, transcription of centromeric non-coding RNAs is tightly controlled and essential for the maintenance of genome integrity [8,10–12]. Transcripts arising from centromeric heterochromatin are now recognized to play important roles in different cellular and developmental contexts [10,13]. For instance, centromeric minor satellite transcripts in mouse and human somatic cells are critical epigenetic determinants of kinetochore function [1,10,13–17].

Transcripts derived from pericentric heterochromatin also play critical roles in chromosome segregation and genome stability during mitosis [2,14,18–20]. However, while there is an increasing understanding of the role of centromeric minor satellite transcripts in the regulation of kinetochore function [21–24], the biological function(s) of non-coding major satellite transcripts derived from pericentromeric heterochromatin are not well understood. We have recently demonstrated that major satellite RNAs are expressed during key stages of mouse oocyte meiosis and remain bound to pericentric heterochromatin domains [25], and expression of pericentric satellite transcripts (HS2, HS3) has also been described in human oocytes [26]. However, the potential role(s) of major satellite transcripts in the regulation of chromosome alignment and meiotic spindle assembly remain unexplored.

Here, we establish a crucial function for pericentric transcription in the maintenance of genome stability during oocyte meiosis. Depletion of major satellite transcripts was associated with striking pericentric heterochromatin structural defects. Importantly, functional ablation induced chromosome alignment errors, and interfered with meiotic maturation potential and spindle organization. Our findings reveal novel functional implications of meiotic major satellite transcripts that involve nascent transcription as well as RNA processing to ensure regulation of pericentric heterochromatin structure, accurate chromosome segregation as well as maintenance of spindle stability.

## 2. Results and discussion

### 2.1. Major satellite transcripts are required for pericentric heterochromatin stability and accurate chromosome segregation during female meiosis

**Figure 1.**
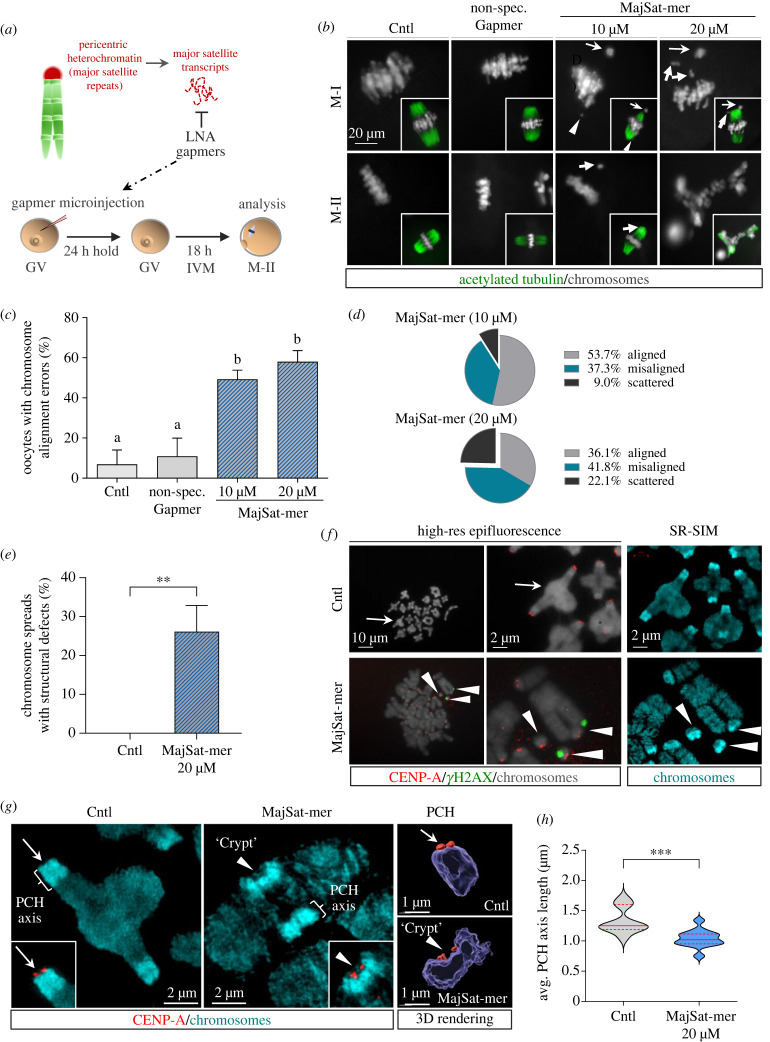
Major satellite transcripts are critical for pericentric heterochromatin stability and chromosome alignment during female meiosis. (*a*) Experimental design schematic. (*b*) Representative images of *in vitro* matured control oocytes (non-injected and non-specific gapmer injected) and oocytes following microinjection of 10 µM or 20 µM major satellite gapmers (MajSat-mer). Metaphase-I (M-I) and metaphase-II (M-II) stage oocytes are stained with DAPI (grey). Spindle microtubules (acetylated tubulin, green) are shown as merged images with DAPI (insets). The position of misaligned bivalents (thin arrows), univalents (bold arrows) and chromosome fragments (arrowheads) at M-I and single chromatids at M-II (bold arrow) is indicated. (*c*) Percent (mean ± std) of total oocytes exhibiting chromosome alignment errors. Data summary from three biological replicates, with greater than 55 oocytes per group. Different superscripts denote statistically significant differences (*p* < 0.05) between groups as analysed by Anova. (*d*) Prevalence of types of alignment errors in 10 µM and 20 µM MajSat-mer injected oocytes. (*e*) Percent (mean ± std) of chromosome spreads exhibiting structural defects. Data summary from 3 biological replicates, with 13–16 spreads per group. **p < 0.01. (*f*) Representative images of chromosome spreads analysed with high-resolution epifluorescence and SR-SIM microscopy. Samples were immunolabelled with CENP-A (red) and the DNA double strand break marker *γ*H2AX (green). DNA is shown in grey (epifluorescence) or cyan (SR-SIM). Arrowheads indicate the position of structural defects (breaks and DNA fragments) in MajSat-mer injected oocytes. (*g*) Superresolution structural illumination microscopy resolves mesoscale changes in pericentric heterochromatin organization in chromosomes from MajSat-mer injected oocytes in the form of PCH hypercondensation and the formation of a concave indentation or ‘crypt’ (arrowhead) partially enclosing fragmented CENP-A signals (red). Imaris 3D-rendering of the indicated PCH domains. (*h*) Average PCH axis measurements (median ± upper and lower quartiles) in control (*n* = 413 PCH domains) and 20 µM MajSat-mer injected oocytes (*n* = 443 PCH domains). ***p < 0.005.

RNA-FISH revealed that major satellite transcripts are expressed at key stages of mouse oocyte meiosis and remain associated with pericentric heterochromatin domains at the germinal vesicle stage, metaphase-I bivalents [25] and metaphase-II chromosomes (electronic supplementary material, figure S1A). Functional ablation of forward and reverse major satellite RNAs (figure 1*a*) using previously validated locked nucleic acid (LNA) gapmers MajSat-mers1/2 [3,8,25] results in a significant (*p* < 0.01) dose-dependent reduction in transcript levels as determined by real-time PCR. By contrast, microinjection of non-specific gapmers showed levels of major satellite transcripts comparable to non-injected controls (electronic supplementary material, figure S1B). Notably, depletion of major satellite RNA with both 10 µM and 20 µM MajSat-mers results in a significant reduction (*p* < 0.001) in the proportion of oocytes reaching the metaphase-II stage following 18 h of *in vitro* maturation (electronic supplementary material, figure S1C), suggesting that major satellite transcripts are required for meiotic progression. *In vitro* matured oocytes also exhibited striking defects in chromosome alignment (46.3% and 63.9% of oocytes) following microinjection with a 10 µM and 20 µM dose, respectively, compared with controls (*p* < 0.01) (figure 1*b–d* and electronic supplementary material, figure S1D). Alignment defects were evident at similar rates in both MajSat-mer injected oocytes that arrested at the M-I stage and oocytes that were able to mature to M-II. We detected misaligned bivalents (thin arrows), formation of univalents (bold arrows) and chromosome fragments (arrowheads) at metaphase-I (M-I) as well as single chromatids at metaphase-II (bold arrow). Microinjection of 20 µM MajSat-mer induced severe misalignment with scattered chromosomes (figure 1*b–d*). Thus, depletion of satellite RNA induces meiotic chromosome segregation errors.

To gain further insight into the effects of satellite RNA depletion, we conducted high-resolution epifluorescence analysis of metaphase-I surface spreads which revealed that microinjection with a 20 µM dose induced a significant increase (*p* < 0.01) in the proportion of oocytes (26.1%) with chromosome structural defects (figure 1*e*). Control oocytes exhibited metaphase-I bivalents with a typical tetrad configuration and CENP-A localization (red) at the kinetochores and no detectable structural defects (figure 1*f*). By contrast, depletion of satellite RNA induced pericentric heterochromatin (PCH) breaks, with formation of chromosome fragments labelled with CENP-A (red) as well as *γ*H2AX signals (green) at the distal PCH boundary, suggesting heterochromatin instability (figure 1*f*; electronic supplementary material, movie S1). These findings expand upon our previous study in which depletion of major satellite transcripts induced abnormal chromocentre organization in GV stage oocytes [25]. Loss of major satellite transcripts is associated with chromosome instability in embryonic stem cells [3]. However, the mechanisms remain unknown. Here, we used superresolution structural illumination microscopy (SR-SIM) to reveal the presence of multiple fragments of DAPI-bright pericentric heterochromatin with unprecedented resolution (figure 1*f*; arrowheads). Notably, we detected significant changes in mesoscale pericentric heterochromatin organization following depletion of major satellite transcripts (figure 1*g*). We used a DAPI intensity threshold for image segmentation of PCH domains in control and MajSat-mer injected oocytes. In control oocytes, chromosome bivalents exhibit large pericentric heterochromatin domains and focused CENP-A signals (red) at the kinetochores, which are positioned towards the surface of the proximal termini of each sister chromatid (figure 1*g*; insets and electronic supplementary material, movie S2). While SR-SIM clearly resolved the typical PCH domain structure of each sister chromatid in control metaphase-I bivalents (electronic supplementary material, figure S1E-F), PCH domains of chromosome spreads from MajSat-mer injected oocytes appeared fused and exhibited striking hypercondensation resulting in the formation of pronounced concavities or ‘crypts’ (figure 1*g*; insets and electronic supplementary material, figure S1E-F). The hypercondensed crypt in PCH domains in MajSat-mer injected oocytes partially enclosed structurally abnormal, and frequently fragmented CENP-A signals (figure 1*g*; insets and electronic supplementary material, movie S3). Quantitative analysis of pericentric heterochromatin domains in control and satellite RNA depleted bivalents revealed a significant reduction (*p* < 0.005) in the average PCH axis length following satellite RNA depletion (figure 1*h*). These results indicate that ablation of major satellite transcripts induced pericentric heterochromatin axial shortening and hypercompaction at metaphase-I and provide evidence of a functional role in the regulation of PCH organization in meiotic chromosomes. Our results also suggest that changes in PCH mesoscale structure may affect centromere topology/function during oocyte maturation and potentially impact chromosome alignment and segregation.

### 2.2. Pericentric heterochromatin instability following major satellite RNA depletion induces meiotic spindle abnormalities

**Figure 2.**
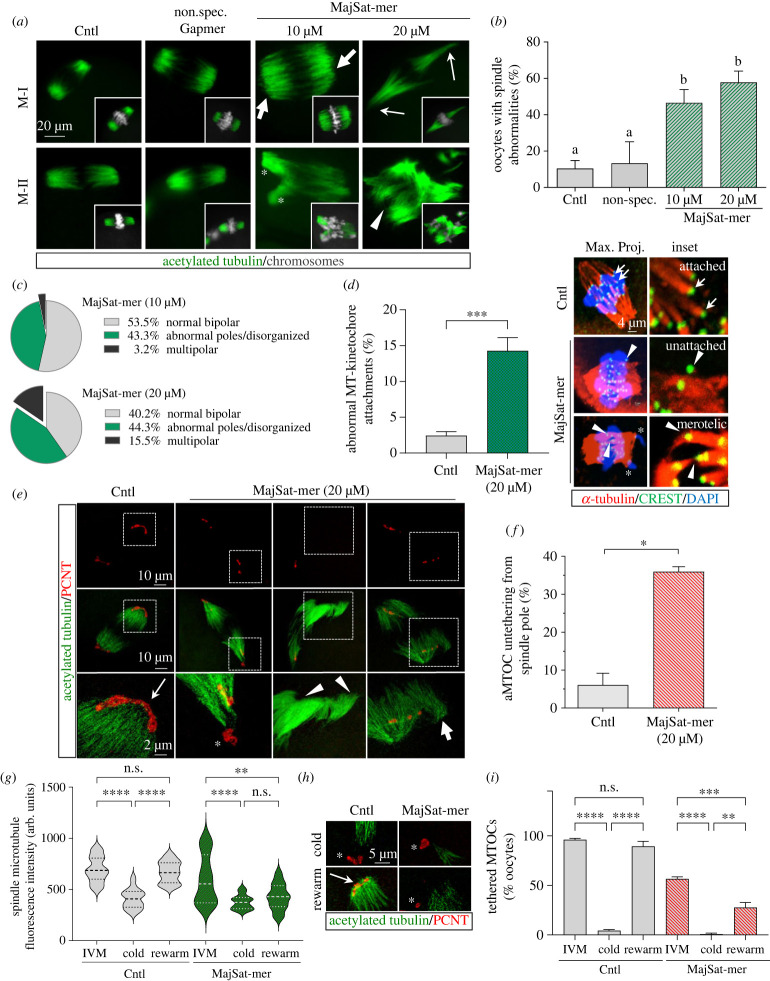
Loss of pericentric heterochromatin stability induces meiotic spindle defects. (*a*) High-resolution analysis of spindle structure in *in vitro* matured oocytes immunolabelled with acetylated tubulin (green). Chromosomes are stained with DAPI (grey) and are shown in insets. Spindle defects in the form of abnormally wide (bold arrows) or narrow (thin arrows) poles, as well as split poles (asterisks) and disorganized spindles (arrowheads). (*b*) Percentage total oocytes (mean ± std) with spindle abnormalities and (*c*) prevalence of spindle defect types observed in oocytes following microinjection of 10 µM and 20 µM major satellite gapmer (MajSat-mer). (*d*) The percent (mean ± std) of abnormal kinetochore–microtubule attachments. ***p < 0.05 in control and MajSat-mer injected oocytes. 3D confocal analysis of kinetochore–microtubule attachments showing representative maximum projection of confocal Z-stacks with chromosomes (blue), microtubules (red), and kinetochores (green) in oocytes (*n* = 20) from control and MajSat-mer injected oocytes. The insets represent maximum projections of relevant optical sections showing normal end-on (arrows), unattached kinetochores as well as abnormal merotelic attachments (arrowheads). (*e*) Representative superresolution structural illumination microscopy (SR-SIM) images of control and MajSat-mer injected oocytes (20 µM). Meiotic spindle microtubules are labelled green, and aMTOCs are stained with PCNT (red). The insets show magnified spindle pole areas. Arrow denotes the typical meiotic aMTOC structure in control oocytes. MajSat-mer injection results in loss of tethering of aMTOCs to the spindle microtubules (asterisk) and may lead to a complete loss of aMTOCs from spindle poles (arrowheads) and/or splaying of microtubule bundles (bold arrow). (*f*) Percent total oocytes showing aMTOC untethering in control and 20 µM MajSat-mer injected oocytes. (*g*) Microtubule fluorescence intensity measurements (median ± upper and lower quartiles) in control and MajSat-mer injected oocytes prior to cold treatment to depolymerize microtubules (IVM), following cold treatment for 10 min at 4°C (cold) and after recovery (5 min, 37°C) from cold treatment (rewarm). (*h*) Representative SR-SIM of spindle poles after cold treatment (cold) and recovery from cold treatment (rewarm) in controls and MajSat-mer injected oocytes. Both experimental groups exhibit depolymerization of microtubules following cold treatment with only cold-stable microtubules (acetylated tubulin, green) detectable. Microtubule regrowth occurs in the majority of control oocytes (thin arrow), while recovery is severely reduced in MajSat-mer injected oocytes. Notably, cold treatment also induces untethering of aMTOCs in both control and MajSat-mer injected oocytes (asterisks, also compare with *e*). However, rewarming rescues aMTOC tethering in controls (arrow), while aMTOCs remain untethered and do not recover in the majority of MajSat-mer injected oocytes (asterisk). (*i*) Percent oocytes (mean ± std) exhibiting tethered aMTOCs. Experiments were conducted with greater than 55 oocytes per group. *p < 0.05, **p < 0.01, ***p < 0.005, ****p < 0.001. Different superscripts denote significant differences (*p* < 0.05) between groups as analysed by Anova.

Next, we sought to determine the effect of chromosome alignment defects induced by loss of satellite RNA on the assembly and/or stability of meiotic spindle structure of *in vitro* matured oocytes. Immunolabelling of acetylated tubulin (green) to visualize stable spindle microtubules confirmed that the majority of control and non-specific gapmer injected oocytes exhibited typical barrel shaped, symmetric and well-organized meiotic spindles (figure 2*a*). By contrast, the proportion of oocytes with spindle defects, including abnormally wide or narrow poles (bold and thin arrows), multipolar (asterisk) or disorganized spindles (arrowhead), increased significantly (*p* < 0.01) in MajSat-mer injected oocytes (figure 2*a–c*). Morphometric analysis of the microtubule arrays near spindle poles revealed a significant decrease (*p* < 0.05) of the mean spindle pole diameter. However, while most oocytes exhibited narrower spindle poles, we also detected a subpopulation of oocytes exhibiting extremely wide poles, indicating a disruption of pole organization. No significant differences were detected in the pole-to-pole spindle length (electronic supplementary material, figure S2A-B). Interestingly, spindle microtubule fluorescence intensity was also highly variable (*p* < 0.001) in MajSat-mer injected oocytes compared to non-injected controls (electronic supplementary material, figure S2C). These results indicate that PCH instability due to loss of major satellite transcripts may indirectly affect microtubule stability, spindle organization and/or microtubule–kinetochore attachments at centromeres. To test these hypotheses, we conducted 3D laser scanning confocal analysis of chromosome–microtubule attachments in *in vitro* matured oocytes (8 h). This analysis revealed a significantly (*p* < 0.005) higher incidence (14.3%) of abnormal kinetochore attachments, including merotelic attachments and unattached kinetochores, in MajSat-mer injected oocytes compared to controls (2.4%) (figure 2*d*). Thus, supporting that depletion of major satellite transcripts interferes with proper kinetochore–microtubule interactions in oocytes, contributing to the high rates of chromosome misalignment observed (figure 1*c*). Moreover, the presence of pericentric heterochromatin fragmentation and the formation of univalents at metaphase-I indicate that abnormal kinetochore-MT attachments lead to the formation of chromosomal breaks during segregation that can be detected by γ-H2AX [27], a bona fide marker of DNA damage (figure 1*f*; electronic supplementary material, figure S1G).

Considering the extensive variability in spindle pole diameter, spindle microtubule fluorescence intensity as well as abnormalities in spindle organization induced by depletion of major satellite transcripts, we next conducted a superresolution analysis of meiotic spindle poles. Meiotic spindle stability in mouse oocytes is influenced, at least in part, by unique acentriolar microtubule organizing centres (aMTOCs) that are comprised of critical scaffolding proteins, such as pericentrin (PCNT) [28,29] and associated factors [30]. Thus, we assessed the aMTOC structure and localization in oocytes with knockdown of major satellite transcripts by immunolabelling *in vitro* matured oocytes with PCNT (red). The majority of control oocytes exhibited a typical ring or C-shaped aMTOC structure (figure 2*e*, thin arrow; electronic supplementary material, figure S2D). By contrast, MajSat-mer injected oocytes showed a significant increase (*p* < 0.01) in aMTOC abnormalities, including tightly clustered or fragmented PCNT (figure 2*e* and electronic supplementary material, figure S2D). Notably, we detected a significant increase (*p* < 0.05) in the percentage of oocytes (36.9%) exhibiting detached PCNT/aMTOCs (figure 2*e*; asterisk) or complete absence of PCNT from meiotic spindle poles (figure 2*e*; arrowheads). In some oocytes, microtubule bundles split and become splayed at the spindle pole (figure 2*e*, bold arrow; electronic supplementary material, figure S2E-F, movie S4) resulting in the formation of multipolar spindles. Moreover, in knockdown oocytes the distance between spindle pole microtubules and the nearest aMTOC varied substantially and showed a significant (*p* < 0.001) increase compared to controls (electronic supplementary material, figure S2G).

To further assess the potential mechanisms of spindle abnormalities, we tested whether major satellite transcript depletion affects the dynamics of microtubule regrowth following depolymerization induced by cold treatment (figure 2*g*,*h*; electronic supplementary material, figure S2H). Incubation at 4°C for 10 min significantly depolymerized spindle microtubules in both control and MajSat-mer injected oocytes, and residual cold-stable microtubules were detectable with similar fluorescence intensity in both groups (figure 2*g*,*h*; electronic supplementary material, figure S2H). Notably, a 5-min incubation at 37°C in pre-warmed media induced microtubule regrowth and typical bipolar spindle assembly in control oocytes (thin arrow), while microtubule regrowth was significantly (*p* < 0.01) reduced in major satellite transcript depleted oocytes (figure 2*g*,*h*; electronic supplementary material, figure S2H). Importantly, cold treatment (4°C for 10 min) induced pronounced aMTOC untethering from spindle poles in both control and MajSat-mer injected oocytes, likely due to loss of stable microtubules (figure 2*h*,*i*, asterisks). However, while almost all control oocytes (89.3%) recovered and exhibited tightly tethered aMTOC following rewarming for 5 min at 37°C, the defect persisted (*p* < 0.01) in the majority (72.4%) of rewarmed MajSat-mer injected oocytes (figure 2*h*,*i*; electronic supplementary material, figure S2H, asterisks). Collectively, these results demonstrate striking effects of loss of pericentric transcripts on spindle pole integrity, microtubule stability and aMTOC tethering to spindle pole microtubules. Importantly, spindle and aMTOC defects were associated with chromosome misalignment in the vast majority of MajSat-mer injected oocytes, suggesting that through the regulation of PCH structure and chromosome–microtubule interactions, major satellite transcripts can also affect oocyte microtubule organizing centres and meiotic spindle organization.

### 2.3. Depletion of major satellite transcripts disrupts spindle pole integrity and polo-like kinase 1 localization

**Figure 3.**
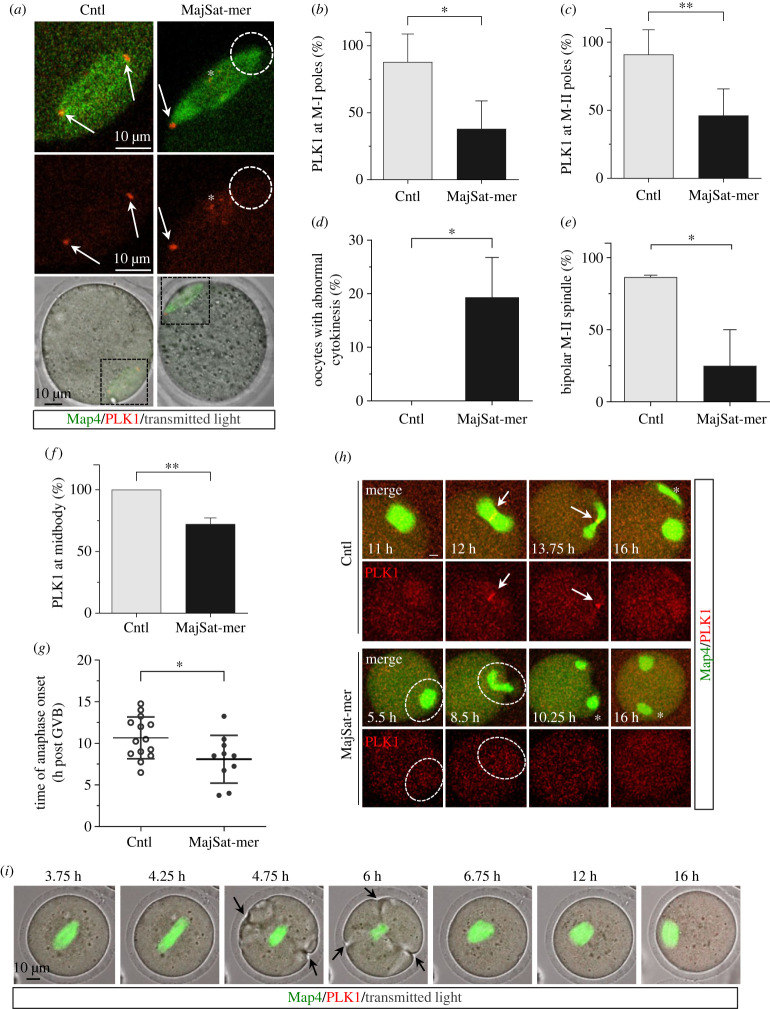
Depletion of major satellite transcripts disrupts PLK1 localization at spindle poles. (*a*) Confocal microscopy images of live oocytes expressing fluorescently labelled microtubule associated protein 4 (MAP4, green) and polo like kinase 1 (PLK1, red). In control oocytes, PLK1 labels meiotic spindle poles (arrows), while MajSat-mer injected oocytes frequently exhibit lack of PLK1 on one or both spindle poles (dashed circles). The asterisk denotes mislocalized PLK1 signals in MajSat-mer injected oocytes. The area of magnification is indicated by the black squares in transmitted light overlays. (*b,c*) Analysis of PLK1 localization to M-I and M-II spindle poles in control and MajSat-mer injected oocytes. (*d*) Percent total oocytes showing abnormal cytokinesis in control and MajSat-mer injected oocytes. (*e*) Rates of bipolar metaphase-II spindle formation. (*f*) Percent oocytes showing PLK1 at the midbody and (*g*) time (mean ± std) of anaphase-I onset in live oocytes. (*h*) Progression from M-I to M-II in representative live cells showing spindle dynamics (MAP4, green) and PLK1 (red, arrows) localization in control and MajSat-mer injected oocytes. Time post GVB is shown. Scale bar of 10 µm. (*i*) Failed polarbody-I extrusion in a major satellite transcript depleted oocyte. Forming cleavage furrows are indicated by arrows. Time post GVB is shown. *p < 0.05; **p < 0.01; not significant (n.s.) compared to the control group. Summary data of 3 biological replicates with greater than 20 oocytes in each group.

Polo-like kinase 1 (PLK1) is essential for the regulation of spindle formation and chromosome congression [31–35]. In mouse oocytes, PLK1 is initially activated at aMTOCs upon meiotic resumption, where it is required for the recruitment of γ-Tubulin and PCNT in order to facilitate microtubule nucleation and stable kinetochore–microtubule attachments [36]. Interestingly, experimental inactivation of the aurora kinase A (AURKA), a PLK1 target, induces aMTOC clustering and abnormal spindle pole focusing [37] similar to the defects observed following major satellite RNA depletion (figure 2*e*). Thus, we imaged live oocytes following microinjection of capped mRNAs encoding recombinant fluorescent PLK1 (red) and the microtubule associated protein MAP4 (green) to monitor the progression of meiosis and subcellular localization of PLK1 over a 16 h time period. PLK1 was detected at the spindle poles in 88% and 91% of control metaphase-I and metaphase-II oocytes, respectively (figure 3*a–c*). However, in the majority of MajSat-mer injected oocytes PLK1 failed to localize to one or both spindle poles, with only 38% and 46% of oocytes exhibiting PLK1 localization at the poles of metaphase-I and metaphase-II spindles, respectively (figure 3*a–c*). Notably, these defects were accompanied by a significant increase (*p* < 0.05) in the proportion of oocytes (19.3%) that failed to complete cytokinesis/extrude a polar body in spite of anaphase-I onset as determined by live cell imaging (figure 3*d*,*i*). Consistent with our previous experiment (figure 2*b*), the majority of MajSat-mer injected oocytes failed to complete meiotic maturation and assemble a bipolar metaphase-II spindle (figure 3*e*). No differences were observed in the dynamics of microtubule nucleation, early spindle assembly and bipolar M-I spindle formation, indicating no overt delay in cell cycle or spindle formation in MajSat-mer injected oocytes (electronic supplementary material, figure S3A). Live cell imaging revealed that in control oocytes PLK1 is localized to the midbody and to the telophase abscission point prior to polar body extrusion (figure 3*f–h*). However, depletion of major satellite RNA is associated with loss of PLK1 localization from a precociously formed midbody and early telophase spindle (dashed circles). Additionally, we observed a significant reduction (*p* < 0.05) in the time of anaphase onset (8.1 ± 2.9 h) in MajSat-mer injected oocytes, including those oocytes that failed to extrude a polar body, compared to controls (10.7 ± 2.5) (figure 3*d*,*g–i*; electronic supplementary material, movie S5). This indicates that loss of PLK1 localization may be associated with premature anaphase onset and disruption of cytokinesis for polar body extrusion and spindle organization, consistent with prior analyses [36,38]. Importantly, our results also indicate that major satellite depletion-induced loss/untethering of aMTOCs is also associated with loss of PLK1 localization to meiotic spindle poles.

### 2.4. Inhibition of transcriptional elongation and processing disrupts meiotic chromosome segregation and spindle pole organization

**Figure 4.**
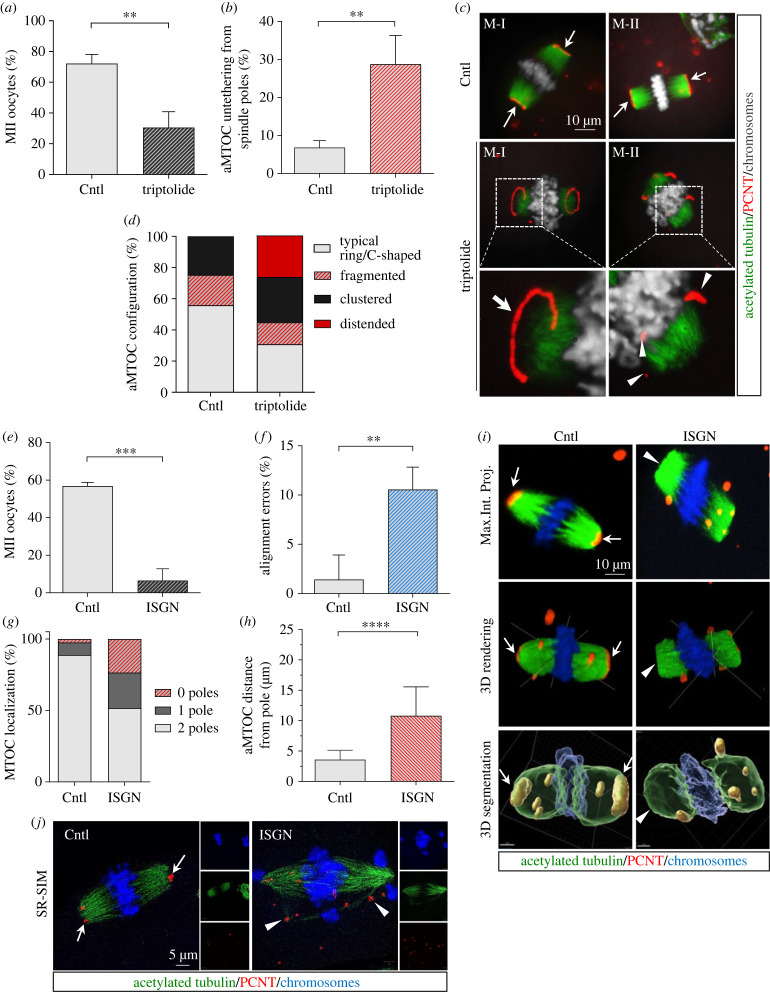
Chromosome alignment and spindle pole organization depend on transcript elongation and processing. (*a*) Effect of inhibition of transcription elongation (50 µM Triptolide) on the percent oocytes that reach M-II (mean ± std) and (*b*) rates of aMTOC untethering (mean ± std) from spindle poles. (*c*) Representative confocal microscopy images of *in vitro* matured oocytes. The position of aMTOCs (PCNT, red) in relation to spindle microtubules (green) in controls (arrows) and oocytes treated with Triptolide, which show aMTOC distension (bold arrow) and fragmentation (arrowheads). DAPI-labelled chromosomes are shown in grey. (*d*) Incidence of different aMTOC configurations observed in control and Triptolide treated oocytes. Summary data of 3 biological replicates, with greater than 60 oocytes in each group. (*e*) Proportion of oocytes (mean ± std) that reach the M-II stage following 18 h IVM in the presence (ISGN, 100 µM) or absence (Cntl) of the splicing inhibitor Isoginkgetin. (*f*) Incidence of alignment errors (mean ± std) in total control and ISGN treated oocytes. Summary data of 3 biological replicates, with greater than 60 oocytes in each group. (*g,h*) Localization of aMTOCs at poles and distance (µm) from spindle poles in control (*n* = 16) and ISGN treated (*n* = 14) oocytes. (*i*) Representative confocal maximum intensity projections (Max.Int.Proj.), 3D renderings and Imaris 3D segmentations of control and ISGN-treated oocytes. Arrows point to aMTOCs (red) at spindle poles in controls, while arrowheads indicate absence of aMTOCs from spindle poles in ISGN treated oocytes. (*j*) Superresolution structured illumination (SR-SIM) microscopy of control and ISGN treated IVM oocytes. aMTOCs are labelled with PCNT (red, arrows). Spindle microtubules are labelled with acetylated tubulin (green), and chromosomes are shown in blue. aMTOC localization at spindle poles is indicated by arrows. Arrowheads point to fragmented aMTOCs that are dissociated from spindle poles. **p < 0.01, ***p < 0.005, ****p < 0.001, compared to the control group.

We previously demonstrated that the transcriptionally engaged isoform of RNA polymerase II (Pol II Ser2ph) localizes, in a transcription-dependent manner, to the inner centromere and the inter-chromatid space of meiotic chromosomes where it partially co-localizes with major satellite transcript foci [25]. This association is maintained despite the global transcriptional shutdown that occurs in preovulatory oocytes [39,40] and persists during meiotic resumption [25], which compelled us to determine the effects of blocking nascent transcripts on meiotic spindle structure by exposing oocytes to the transcription initiation and elongation inhibitor Triptolide [41]. Exposure to 50 µM Triptolide for 18 h during meiotic maturation significantly reduced (*p* < 0.01) the proportion of oocytes (30.5%) that reached the metaphase-II stage, compared with controls (72.2%) (figure 4*a*). Moreover, we detected a significant increase (*p* < 0.01) in the proportion of oocytes (28.8%) that exhibited untethering of aMTOCs from the spindle poles (figure 4*b*). While the majority of control oocytes exhibit a bipolar spindle and ring- or C-shaped aMTOCs at spindle poles (figure 4*c*,*d*; arrow), Triptolide exposure induced the formation of highly distended PCNT rings (red, bold arrow) or the loss of spindle pole integrity with PCNT fragmentation (figure 4*c*, arrowheads and electronic supplementary material, figure S3B). These results indicate that despite the global repression of transcription that ensues prior to germinal vesicle breakdown [39,42], a basal level of nascent transcription from major satellites during oocyte meiosis still occurs and is required for proper chromosome segregation and spindle pole integrity. Importantly, Triptolide exposure induced similar meiotic spindle pole defects as observed following depletion of major satellite transcripts using specific locked nucleic acid gapmers (figure 2). Together with the localization of Pol II Ser2ph at the inner centromere, our results provide evidence that nascent transcription of major satellite RNAs and possibly other non-coding RNAs is required to regulate spindle stability and accurate chromosome and microtubule interactions in otherwise transcriptionally quiescent oocytes.

Moreover, to determine whether processing of non-coding RNAs is required for oocyte maturation, we exposed maturing oocytes to the RNA splicing inhibitor Isoginkgetin (ISGN) [43]. Treatment with 100 µM ISGN during oocyte maturation significantly reduced (*p* < 0.005) the proportion of oocytes (6.5%) that progressed to metaphase-II, compared with controls (56.9%), and increased the proportion of oocytes (10.6%) with chromosome alignment defects (*p* < 0.01) (figure 4*e*,*f*). Importantly, 3D renderings of the meiotic spindle revealed detachment of PCNT from one or both poles and fragmentation/untethering of this essential aMTOC scaffolding protein associated with the loss of spindle pole integrity (figure 4*g–i*). Superresolution (SR-SIM) analysis demonstrated severe PCNT fragmentation as well as abnormal chromosome alignment (figure 4*j*). Notably, simultaneous treatment with 100 nM Reversine, an inhibitor of the spindle assembly checkpoint (SAC), significantly increased (*p* < 0.01) the proportion of oocytes reaching the metaphase-II stage (electronic supplementary material, figure S3C), suggesting that meiotic arrest is likely due to abnormal centromere/kinetochore complex–microtubule interactions. These results indicate that RNA processing during oocyte meiosis is required for oocyte meiotic maturation and meiotic spindle stability.

**Figure 5.**
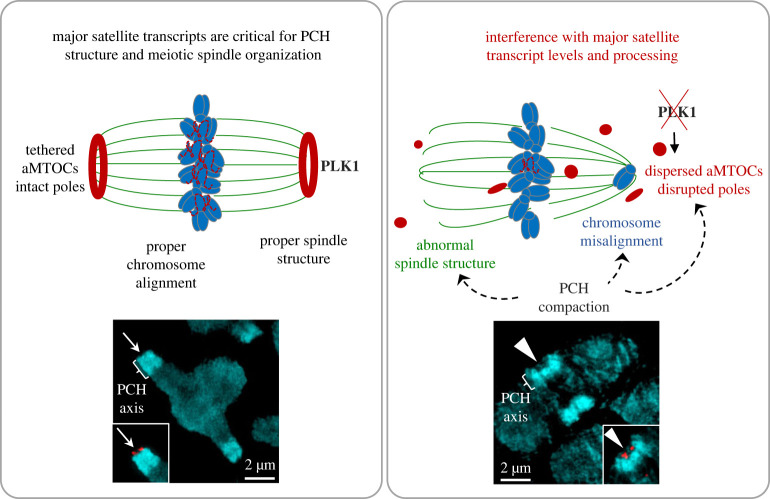
Proposed model for the functional roles of pericentric major satellite RNA during oocyte meiosis. Pericentric major satellite RNAs play functional roles in regulating chromosome stability, meiotic progression as well as spindle stability. Depletion of major satellite RNA induces pericentric heterochromatin (PCH) compaction at metaphase-I, centromere breaks and significant chromosome misalignment. The chromosome errors are associated with disruption of spindle pole integrity, abnormal aMTOC organization with displacement from the spindle poles and are associated with loss of PLK1.

Collectively, our results provide evidence that the expression and chromosomal localization of pericentric major satellite RNA play functional roles in regulating meiotic progression and spindle stability and suggest a working model (figure 5) in which depletion of major satellite RNA induced pericentric heterochromatin compaction at metaphase-I, centromere breaks and significant chromosome misalignment. The chromosome errors were associated with MT-kinetochore attachment errors, disruption of spindle stability and pole integrity, multipolar spindles and altered aMTOC organization and/or displacement. PCNT fragmentation and compromised spindle pole integrity was associated with loss of PLK1. Notably, inhibition of transcriptional elongation or RNA splicing induced similar meiotic spindle and chromosome errors, suggesting that nascent transcription and processing of major satellite transcripts during oocyte meiosis are required to regulate heterochromatin structure, contributing to chromosome alignment and spindle pole organization and are, thus, necessary for accurate chromosome segregation.

## 3. Conclusion

Elegant studies have recently demonstrated a critical role for major satellite non-coding RNAs in heterochromatin formation and maintenance of genome integrity in somatic [14,18] and more recently in ES cells [3]. However, the role of major satellite RNA during oocyte meiosis remained to be determined. Our studies support a critical function for major satellite RNA in the regulation of mesoscale pericentric heterochromatin organization at metaphase-I. Chromatin structural modifications of the inner centromere are known to affect the tension sensing mechanisms at the mitotic spindle and may affect both kinetochores and pericentric heterochromatin domains [44]. Our studies provide evidence that by playing a role in regulating chromosome–microtubule interactions, major satellite RNA is critical both for accurate chromosome alignment and meiotic spindle organization. Thus, the functional interplay between centromeric domains and centrosomes recently uncovered in mitotic cells, whereby abnormal centromere function can also disrupt mitotic spindle pole integrity [45], is seemingly conserved during oocyte meiosis. Studies have shown that major and minor satellite transcripts are also critical for chromosome segregation during male meiosis [46,47], and that minor satellite RNAs are required to regulate kinetochore–microtubule interactions in mouse oocytes [48]. These findings support that both major and minor satellite RNAs play critical functional roles in regulating meiotic progression in oocytes. Moreover, emerging evidence indicates that, similar to mitotic cells [2,18–20], centromere function in gametes requires stringent regulation of pericentric transcript levels to maintain chromosome stability, as excessive accumulation of major satellite RNAs in MIWI or Dicer mutant spermatocytes [46,47], and in LSH mutant oocytes is also associated with meiotic aneuploidy [9,25].

Our data support a proposed model in which PCH erosion following depletion of major satellite RNA likely affects centromere function through pericentric heterochromatin axial shortening and structural defects that may lead to abnormal centromere topology and fragmentation of essential kinetochore proteins such as CENP-A (figure 5). Importantly, we reveal novel functional implications of meiotic major satellite transcripts through nascent transcription as well as processing of meiotic RNAs to ensure not only maintenance of chromatin structure and chromosome alignment, but also by affecting PCH topological interactions required for spindle stability and pole integrity.

## 4. Experimental procedures

### 4.1. Animals

All mice were housed at a constant temperature (24–26°C) and a controlled light cycle (12 h light/dark), with food and water provided ad libitum. Animal use protocols were approved by the Institutional Animal Care and Use Committee (IACUC) at the University of Georgia (Athens, GA, USA), and experiments were conducted in accordance with the specified guidelines. All experiments used oocytes collected from C57Bl6/DBA hybrid females 20 to 24 dpp.

### 4.2. Oocyte collection and culture

Preovulatory oocytes were collected 48 h after administration of 5 IU pregnant mare serum gonadotrophin (EMD Biosciences, La Jolla, CA) to promote follicle growth. The oocytes were recovered from ovarian follicles by needle puncture and transferred to minimal essential medium (MEM) supplemented with 3 mg ml^−1^ bovine serum albumin and 10 µM milrinone (Sigma Aldrich, St Louis, MO). Surrounding granulosa cells were removed by gentle pipetting in MEM/BSA followed by several rinses in fresh media. For *in vitro* maturation, oocytes were cultured at 37°C in MEM/BSA supplemented with 5% FBS (Atlanta Biologicals, Flowery Branch, GA) for 18 h under 5% CO_2_, 5% O_2_ and N_2_. Oocytes were then fixed in prewarmed 4% PFA, 0.25% Triton X-100 for 30min at 37°C before serial washes and blocking in block buffer (5% FBS, 0.05% Triton X-100) overnight at 4°C. Chromosome spreads were prepared following chemical removal of the zona pellucida using Tyrode's solution (Sigma Aldrich). Zona free oocytes were placed on glass slides and lysed in a fixative solution of 1% PFA, 0.15% Triton X-100 before airdrying [5].

### 4.3. Microinjection

The role of major satellite RNAs was assessed by functional knockdown of transcripts using LNA-DNA major satellite gapmers (Qiagen) described previously [8,25]. GV stage oocytes were denuded and microinjected with 10 pl of a 10 µM or 20 µM cocktail of forward and reverse LNA-DNA major satellite gapmers using an Eppendorf micromanipulator on a Nikon inverted microscope, while control oocytes were in parallel injected with non-specific LNA-DNA GFP gapmers (non-specific Gapmer) or remained non-injected (Cntl). Oocytes were then cultured for 24 h to allow for the knockdown of transcripts, before *in vitro* maturation for 18 h and fixation in 4% PFA, 0.05% Tx-100 for 30 min at 37 °C.

### 4.4. RNA extraction and quantitative expression analysis

For qPCR assays, total RNA was isolated from groups (*n* = 50) of denuded oocytes using the RNeasy Micro kit (Qiagen) and subsequently reverse transcribed using Superscript III First Strand Synthesis System for RT-PCR (Invitrogen) with random hexamer primers. Major satellite-specific transcripts were detected using oligonucleotides with the sequence Fwd: 5′-GACGACTTGAAAAATGACGAAATC-3′ and Rev: 5′-CATATTCCAGGTCCTTCAGTGTGC-3′ [49], and expression was normalized using β-actin as a housekeeping control (Qiagen, PPM02945B-200).

### 4.5. Immunofluorescence

Whole mount oocytes were fixed and immunolabelled with specific antibodies as previously described [9,50] to detect PCNT (1 : 1000, PRB432C, Covance, Princeton, NJ) and acetylated α-Tubulin (1 : 1000, T6793, Sigma Aldrich). In brief, fixed oocytes were incubated with the primary antibodies for 1 h at 37°C, then washed and incubated (1 h) at 37°C with specific Alexa Fluor conjugated 488 or 555 secondary antibodies (1 : 1000, A11070, A21425, A11017, A21430, Life Technologies, Eugene, OR). After a final wash, the oocytes were transferred onto glass slides and overlaid with mounting medium (VectaShield, Vector Laboratories, Burlingame, CA) containing DAPI (4′,6-diamidino-2-phenylindole) to counterstain the DNA. Fluorescence was assessed using a Leica DMRE upright fluorescent microscope with imaging software (Leica Microsystems). When indicated, three-dimensional (3D) imaging and reconstruction of meiotic spindles was performed on oocytes in suspension using a Nikon Eclipse Ti-U/D-Eclipse C1 laser scanning confocal microscope equipped with a 40× objective lens following sequential (frame lambda) excitation of GFP fusion proteins with a 488 nm Coherent Sapphire laser and RFP fusion proteins with a 561 Coherent Sapphire laser. Image acquisition was conducted using EZ-C1 software (Nikon) with a step size of 1µm and a *Z*-stack range of 50 µm. Imaging data were subsequently analysed by maximum intensity and 3D reconstructions using NIH Elements software (Nikon) as well as using IMARIS (Oxford Instruments) for quantitative analysis.

Surface spreads of chromosomes were immunostained using specific antibodies against CENP-A (rabbit polyclonal, 1 : 200, C51A7, Cell Signaling Technologies) and *γ*H2AX (mouse polyclonal, 1 : 500, ab26350, abcam). Primary antibodies were detected with specific Alexa Fluor conjugated 488 or 555 secondary antibodies (1 : 1000, A11070, A21425, A11017, A21430, Life Technologies, Eugene, OR).

Oocytes and chromosome spreads were also assessed by high resolution structured illumination microscopy (SR-SIM) using a Zeiss Elyra S1 system equipped with a 100× oil immersion lens, and ZEN 2011 software with a SIM analysis module for image acquisition at the Biomedical Microscopy Core (BMC) facility, University of Georgia.

### 4.6. Microtubule stability regrowth analysis

Spindle microtubule regrowth following depolymerization with cold treatment was assessed in control and MajSat-mer injected oocytes as previously described [28,37]. *In vitro* matured (18 h) oocytes were randomly allocated to three experimental groups consisting of (i) a non-cold treated ‘IVM’ group, (ii) a ‘cold’ treated group and (iii) a cold treated then recovered group termed ‘rewarm’. Oocytes from the IVM group were fixed for immunofluorescence analysis immediately following the 18 h IVM culture, while the remaining oocytes were placed in cold M2 medium (Sigma) at 4°C for 10 minutes to depolymerize the spindle microtubules. After cold treatment, one group of oocytes was immediately fixed for analysis, while the remaining group was transferred to fresh pre-warmed MEM medium at 37°C for microtubule regrowth, and then fixed for immunofluorescence analysis at 5 minutes post warming. All oocytes were immunolabelled with anti-PCNT and anti-acetylated α-tubulin antibodies, as described above. Spindle microtubule organization was assessed using an upright fluorescent microscope with imaging software (Leica Microsystems) and by SR-SIM using a Zeiss Elyra S1 system equipped with a 100× oil immersion lens, and ZEN 2011 software with a SIM analysis module.

### 4.7. Assessment of kinetochore–microtubule attachments

Oocytes were processed for kinetochore–microtubule attachment analysis, as described previously [29]. Oocytes at 6.5 h post GVB were cold treated at 4°C for 10 min to depolymerize the spindle microtubules and fixed immediately in 4% PFA. Prior to immunochemistry, the oocytes were permeabilized in PBS supplemented with 0.1% Triton X-100 and 0.3% BSA for 15 minutes at room temperature. Oocytes were then immunolabelled as described above using Human CREST autoimmune serum (1 : 500 dilution of Nuclear ANA-Centromere Autoantibody, Cortex Biochem, Concord, MA, CS1058) together with anti-α-tubulin (1 : 1000; Sigma, T6793, clone 6-11B-1) overnight at 4°C, to label kinetochores and cold-stable spindle microtubules (MTs). After the final wash, the cells were transferred into wash buffer containing DAPI to counterstain the DNA and kept in suspension. The entire meiotic spindle was then visualized three-dimensionally by confocal microscopy using a Nikon Eclipse Ti-U/D-Eclipse C1 laser scanning confocal microscope equipped with a 40× oil immersion lens following sequential (frame lambda) excitation with a 488 and 561 nm Coherent Sapphire laser, to detect MTs and kinetochores, respectively. Image acquisition was conducted using EZ-C1 software (Nikon) with a step size of 0.3 µm and a Z-stack range of 11–15 µm. Confocal Z-stacks were subsequently analysed using NIH Elements software (Nikon) to classify kinetochore–microtubule attachments Z-frame by Z-frame.

### 4.8. RNA-FISH

RNA-FISH experiments were conducted according to previously described procedures [25]. Briefly, zona-free GV or metaphase stage oocytes were permeabilized with a solution of 0.5% Triton X-100 in DEPC-PBS for 2 min at room temperature before washing three times in 2× SSC. A Cy5 labelled major satellite-specific FISH probe (Cambio Ltd) was denatured at 80°C for 8 min, cooled to 37 °C and applied onto the surface-spread samples. Hybridization was carried out overnight at 37°C in a humidified chamber. Post-hybridization washes were conducted in 1× SSC in DEPC-H_2_O for 5 min at room temperature before mounting in Vectashield plus DAPI.

### 4.9. Live-cell imaging

Live-cell imaging was used to compare meiotic spindle assembly during meiotic division in real time between control and MajSat-mer injected oocytes. Time-lapse image acquisition was performed as previously described [29] following co-microinjection of a capped messenger RNA (cRNA) cocktail encoding PLK1–mCherry and MAP4–EGPF fusion proteins with or without MajSat-mers into the cytoplasm of fully grown prophase-I arrested (GV-stage) oocytes in order to visualize the PLK1 (red) and MTs (green), respectively. Capped mRNAs were synthesized from vectors pGEMHE-EGFP-MAP4 and pCS2-PLK1-mCherry (Addgene) using the mMESSAGE mMACHINE Kit (ThermoFisher) as previously described [5]. Following cRNA microinjection, the oocytes were cultured at 37°C overnight in MEM/BSA with 10 µM milrinone to allow recombinant protein expression during prophase-I arrest. The next day, the oocytes were washed in fresh medium with no milrinone and transferred to a 200 µl microdrop of MEM/BSA medium supplemented with 5% FBS under mineral oil within an environmental chamber (Tokai Hit, Fujinomiya, Japan). The temperature was maintained at 37°C in an atmosphere of 5% CO_2_, 5% O_2_ and balanced N_2_. The dynamics of spindle assembly and stability as well as the localization of PLK1 were monitored for 16 h by time-lapse microscopy at 15-min intervals using a Nikon Eclipse Ti-U/D-Eclipse C1 laser scanning confocal microscope equipped with a 40× objective lens following sequential (frame lambda) excitation of GFP fusion proteins with a 488 nm Coherent Sapphire laser and RFP fusion proteins with a 561 Coherent Sapphire laser. Image acquisition was conducted using EZ-C1 software (Nikon) with a step size of 2 µm and a Z-stack range of 100 µm. Live-cell imaging data were subsequently analysed by maximum intensity and 3D reconstructions by using NIH Elements software (Nikon).

### 4.10. Inhibitors of transcript processing and transcriptional elongation

For some analyses, oocytes were treated with 100 µM Isoginkgetin (416154, Sigma Aldrich) alone, co-treated with 100 µM Isoginkgetin and 100 nM Reversine (R3904, Sigma Aldrich) or treated with vehicle solvent alone (DMSO) to assess the effects of interference with RNA splicing on meiotic maturation. In other experiments, oocytes were treated with 50 µM Triptolide (T3652, Sigma Aldrich) to gain insight into the role of transcriptional elongation during meiotic maturation. Control group oocytes were incubated in media containing the appropriate concentration of solvent (DMSO) only under similar conditions. All cultures were maintained at 37°C in MEM/BSA under 5% CO_2_, 5% O_2_ and N_2_. Following culture, oocytes were immediately fixed for immunofluorescence analysis to assess the spindle microtubules, aMTOC components and chromosome configurations.

### 4.11. Quantification and statistical analysis

All data are presented as the mean values (± std) from at least three independent experimental replicates with at least 40 oocytes per sample, unless otherwise noted. The data were analysed by one-way or two-way ANOVA, Mann–Whitney test or *t*-test for comparison among groups depending on D'Agostino and Pearson normality testing, using the GraphPad Prism 9 software. Differences were considered significant when *p* < 0.05. Asterisks denote the following: *p < 0.05; **p < 0.01; ***p < 0.005; ****p < 0.001, and different superscripts indicate significant differences.

## Data Availability

All the data are available from the corresponding author and can be accessed with a valid reason. Supplementary material is available online [51].

## References

[1] Allshire RC, Madhani HD. 2018 Ten principles of heterochromatin formation and function. Nat. Rev. Mol. Cell Biol. **19**, 229-244. (10.1038/nrm.2017.119) 29235574 PMC6822695

[2] Janssen A, Colmenares SU, Karpen GH. 2018 Heterochromatin: guardian of the genome. Annu. Rev. Cell Dev. Biol. **34**, 265-288. (10.1146/annurev-cellbio-100617-062653) 30044650

[3] Novo CL et al. 2022 Satellite repeat transcripts modulate heterochromatin condensates and safeguard chromosome stability in mouse embryonic stem cells. Nat. Commun. **13**, 3525. (10.1038/s41467-022-31198-3) 35725842 PMC9209518

[4] Probst AV, Almouzni G. 2011 Heterochromatin establishment in the context of genome-wide epigenetic reprogramming. Trends Genet. **27**, 177-185. (10.1016/j.tig.2011.02.002) 21497937

[5] Baumann C, Viveiros MM, De La Fuente R. 2010 Loss of maternal ATRX results in centromere instability and aneuploidy in the mammalian oocyte and pre-implantation embryo. PLoS Genet. **6**, e1001137. (10.1371/journal.pgen.1001137) 20885787 PMC2944790

[6] Lachner M, O'Carroll D, Rea S, Mechtler K, Jenuwein T. 2001 Methylation of histone H3 lysine 9 creates a binding site for HP1 proteins. Nature **410**, 116-120. (10.1038/35065132) 11242053

[7] Bannister AJ, Zegerman P, Partridge JF, Miska EA, Thomas JO, Allshire RC, Kouzarides T. 2001 Selective recognition of methylated lysine 9 on histone H3 by the HP1 chromo domain. Nature **410**, 120-124. (10.1038/35065138) 11242054

[8] Probst AV, Okamoto I, Casanova M, El Marjou F, Le Baccon P, Almouzni G. 2010 A strand-specific burst in transcription of pericentric satellites is required for chromocenter formation and early mouse development. Dev. Cell **19**, 625-638. (10.1016/j.devcel.2010.09.002) 20951352

[9] De La Fuente R, Baumann C, Viveiros MM. 2015 ATRX contributes to epigenetic asymmetry and silencing of major satellite transcripts in the maternal genome of the mouse embryo. Development **142**, 1806-1817. (10.1242/dev.118927) 25926359 PMC4440925

[10] Biscotti MA, Canapa A, Forconi M, Olmo E, Barucca M. 2015 Transcription of tandemly repetitive DNA: functional roles. Chromosome Res. **23**, 463-477. (10.1007/s10577-015-9494-4) 26403245

[11] Hartley G, O'Neill RJ. 2019 Centromere repeats: hidden gems of the genome. Genes **10**, 223. (10.3390/genes10030223) 30884847 PMC6471113

[12] Arunkumar G, Melters DP. 2020 Centromeric transcription: a conserved Swiss-Army knife. Genes (Basel) **11**, 911. (10.3390/genes11080911) 32784923 PMC7463856

[13] Chan FL, Marshall OJ, Saffery R, Won Kim B, Earle E, Choo KHA, Wong LH. 2012 Active transcription and essential role of RNA polymerase II at the centromere during mitosis. Proc. Natl Acad. Sci. USA **109**, 1979-1984. (10.1073/pnas.1108705109) 22308327 PMC3277563

[14] Velazquez Camacho O et al. 2017 Major satellite repeat RNA stabilize heterochromatin retention of Suv39h enzymes by RNA-nucleosome association and RNA:DNA hybrid formation. Elife **6**, e25293. (10.7554/eLife.25293) 28760199 PMC5538826

[15] Bouzinba-Segard H, Guais A, Francastel C. 2006 Accumulation of small murine minor satellite transcripts leads to impaired centromeric architecture and function. Proc. Natl Acad. Sci. USA **103**, 8709-8714. (10.1073/pnas.0508006103) 16731634 PMC1482643

[16] Bobkov GOM, Gilbert N, Heun P. 2018 Centromere transcription allows CENP-A to transit from chromatin association to stable incorporation. J. Cell Biol. **217**, 1957-1972. (10.1083/jcb.201611087) 29626011 PMC5987708

[17] Nakano M, Cardinale S, Noskov VN, Gassmann R, Vagnarelli P, Kandels-Lewis S, Larionov V, Earnshaw WC, Masumoto H. 2008 Inactivation of a human kinetochore by specific targeting of chromatin modifiers. Dev. Cell **14**, 507-522. (10.1016/j.devcel.2008.02.001) 18410728 PMC2311382

[18] Kishikawa T, Otsuka M, Yoshikawa T, Ohno M, Ijichi H, Koike K. 2016 Satellite RNAs promote pancreatic oncogenic processes via the dysfunction of YBX1. Nat. Commun. **7**, 13006. (10.1038/ncomms13006) 27667193 PMC5052683

[19] Smurova K, De Wulf P. 2018 Centromere and pericentromere transcription: roles and regulation…in sickness and in health. Front. Genet. **9**, 674. (10.3389/fgene.2018.00674) 30627137 PMC6309819

[20] Zhu Q et al. 2018 Heterochromatin-encoded satellite RNAs induce breast cancer. Mol. Cell **70**, 842-853.e7. (10.1016/j.molcel.2018.04.023) 29861157 PMC6545586

[21] Rošić S, Köhler F, Erhardt S. 2014 Repetitive centromeric satellite RNA is essential for kinetochore formation and cell division. J. Cell Biol. **207**, 335-349. (10.1083/jcb.201404097) 25365994 PMC4226727

[22] Bury L, Moodie B, Ly J, Mckay LS, Miga KHH, Cheeseman IM. 2020 Alpha-satellite RNA transcripts are repressed by centromere–nucleolus associations. Elife **9**, e59770. (10.7554/eLife.59770) 33174837 PMC7679138

[23] Chen Y, Zhang Q, Teng Z, Liu H. 2021 Centromeric transcription maintains centromeric cohesion in human cells. J. Cell Biol. **220**, e202008146. (10.1083/jcb.202008146) 33881484 PMC8065269

[24] McNulty SM, Sullivan LL, Sullivan BA. 2017 Human centromeres produce chromosome-specific and array-specific alpha satellite transcripts that are complexed with CENP-A and CENP-C. Dev. Cell **42**, 226-240.e6. (10.1016/j.devcel.2017.07.001) 28787590 PMC5568664

[25] Baumann C, Ma W, Wang X, Kandasamy MK, Viveiros MM, De La Fuente R. 2020 Helicase LSH/Hells regulates kinetochore function, histone H3/Thr3 phosphorylation and centromere transcription during oocyte meiosis. Nat. Commun. **11**, 4486. (10.1038/s41467-020-18009-3) 32900989 PMC7478982

[26] Dobrynin MA et al. 2020 Human pericentromeric tandemly repeated DNA is transcribed at the end of oocyte maturation and is associated with membraneless mitochondria-associated structures. Sci. Rep. **10**, 19634. (10.1038/s41598-020-76628-8) 33184340 PMC7665179

[27] Muñoz DP, Kawahara M, Yannone SM. 2013 An autonomous chromatin/DNA-PK mechanism for localized DNA damage signaling in mammalian cells. Nucleic Acids Res. **41**, 2894-2906. (10.1093/nar/gks1478) 23325849 PMC3597672

[28] Ma W, Viveiros MM. 2014 Depletion of pericentrin in mouse oocytes disrupts microtubule organizing center function and meiotic spindle organization. Mol. Reprod. Dev. **81**, 1019-1029. (10.1002/mrd.22422) 25266793 PMC4229429

[29] Baumann C, Wang X, Yang L, Viveiros MM. 2017 Error-prone meiotic division and subfertility in mice with oocyte-conditional knockdown of pericentrin. J. Cell Sci. **130**, 1251-1262. (10.1242/jcs.196188) 28193732 PMC6518315

[30] Ma W, Baumann C, Viveiros MM. 2010 NEDD1 is crucial for meiotic spindle stability and accurate chromosome segregation in mammalian oocytes. Dev. Biol. **339**, 439-450. (10.1016/j.ydbio.2010.01.009) 20079731

[31] Severance AL, Latham KE. 2017 PLK1 regulates spindle association of phosphorylated eukaryotic translation initiation factor 4E-binding protein and spindle function in mouse oocytes. Am. J. Physiol. Cell Physiol. **313**, C501-C515. (10.1152/ajpcell.00075.2017) 28794108 PMC5792166

[32] Alfaro E, López-Jiménez P, González-Martínez J, Malumbres M, Suja JA, Gómez R. 2021 PLK1 regulates centrosome migration and spindle dynamics in male mouse meiosis. EMBO Rep. **22**, e51030. (10.15252/embr.202051030) 33615693 PMC8025030

[33] Little TM, Jordan PW. 2020 PLK1 is required for chromosome compaction and microtubule organization in mouse oocytes. Mol. Biol. Cell **31**, 1206-1217. (10.1091/mbc.E19-12-0701) 32267211 PMC7353151

[34] Tong C, Fan H-Y, Lian L, Li S-W, Chen D-Y, Schatten H, Sun Q-Y. 2002 Polo-like kinase-1 is a pivotal regulator of microtubule assembly during mouse oocyte meiotic maturation, fertilization, and early embryonic mitosis. Biol. Reprod. **67**, 546-554. (10.1095/biolreprod67.2.546) 12135894

[35] Addis Jones O, Tiwari A, Olukoga T, Herbert A, Chan K-L. 2019 PLK1 facilitates chromosome biorientation by suppressing centromere disintegration driven by BLM-mediated unwinding and spindle pulling. Nat. Commun. **10**, 2861. (10.1038/s41467-019-10938-y) 31253795 PMC6599003

[36] Solc P et al. 2015 Multiple requirements of PLK1 during mouse oocyte maturation. PLoS One **10**, e0116783. (10.1371/journal.pone.0116783) 25658810 PMC4319955

[37] Wang X, Baumann C, De La Fuente R, Viveiros MM. 2020 CEP215 and AURKA regulate spindle pole focusing and aMTOC organization in mouse oocytes. Reproduction **159**, 261-274. (10.1530/REP-19-0263) 31895686 PMC7030940

[38] Pomerantz Y, Elbaz J, Ben-Eliezer I, Reizel Y, David Y, Galiani D, Nevo N, Navon A, Dekel N. 2012 From ubiquitin-proteasomal degradation to CDK1 inactivation: requirements for the first polar body extrusion in mouse oocytes. FASEB J. **26**, 4495-4505. (10.1096/fj.12-209866) 22859367

[39] Bouniol-Baly C, Hamraoui L, Guibert J, Beaujean N, Szöllösi MS, Debey P. 1999 Differential transcriptional activity associated with chromatin configuration in fully grown mouse germinal vesicle oocytes. Biol. Reprod. **60**, 580-587. (10.1095/biolreprod60.3.580) 10026102

[40] De La Fuente R, Viveiros MM, Burns KH, Adashi EY, Matzuk MM, Eppig JJ. 2004 Major chromatin remodeling in the germinal vesicle (GV) of mammalian oocytes is dispensable for global transcriptional silencing but required for centromeric heterochromatin function. Dev. Biol. **275**, 447-458. (10.1016/j.ydbio.2004.08.028) 15501230

[41] Vispé S et al. 2009 Triptolide is an inhibitor of RNA polymerase I and II–dependent transcription leading predominantly to down-regulation of short-lived mRNA. Mol. Cancer Therapeut. **8**, 2780-2790. (10.1158/1535-7163.MCT-09-0549) 19808979

[42] De La Fuente R. 2006 Chromatin modifications in the germinal vesicle (GV) of mammalian oocytes. Dev. Biol. **292**, 1-12. (10.1016/j.ydbio.2006.01.008) 16466710

[43] O'Brien K, Matlin AJ, Lowell AM, Moore MJ. 2008 The biflavonoid isoginkgetin is a general inhibitor of Pre-mRNA splicing. J. Biol. Chem. **283**, 33 147-33 154. (10.1074/jbc.M805556200) PMC2586251 18826947

[44] Haase J, Stephens A, Verdaasdonk J, Yeh E, Bloom K. 2012 Bub1 kinase and Sgo1 modulate pericentric chromatin in response to altered microtubule dynamics. Curr. Biol. **22**, 471-481. (10.1016/j.cub.2012.02.006) 22365852 PMC3311747

[45] Gemble S et al. 2019 Centromere dysfunction compromises mitotic spindle pole integrity. Curr. Biol. **29**, 3072-3080.e5. (10.1016/j.cub.2019.07.052) 31495582

[46] Yadav RP et al. 2020 DICER regulates the expression of major satellite repeat transcripts and meiotic chromosome segregation during spermatogenesis. Nucleic Acids Res. **48**, 7135-7153. (10.1093/nar/gkz1163) 32484548 PMC7367195

[47] Hsieh CL, Xia J, Lin H. 2020 MIWI prevents aneuploidy during meiosis by cleaving excess satellite RNA. EMBO J. **39**, e103614. (10.15252/embj.2019103614) 32677148 PMC7429737

[48] Wu T, Lane SI, Morgan SL, Tang F, Jones KT. 2021 Loss of centromeric RNA activates the spindle assembly checkpoint in mammalian female meiosis I. J. Cell Biol. **220**, e202011153. (10.1083/jcb.202011153) 34379093 PMC8360762

[49] Lehnertz B et al. 2003 Suv39h-mediated histone H3 lysine 9 methylation directs DNA methylation to major satellite repeats at pericentric heterochromatin. Curr. Biol. **13**, 1192-1200. (10.1016/S0960-9822(03)00432-9) 12867029

[50] Baumann C, Viveiros MM. 2015 Meiotic spindle assessment in mouse oocytes by siRNA-mediated silencing. J. Vis. Exp. **11**, 53586. (10.3791/53586) PMC4692649 26485537

[51] Baumann C, Zhang X, Viveiros MM, De La Fuente R. 2023 Pericentric major satellite transcription is essential for meiotic chromosome stability and spindle pole organization. Figshare. (10.6084/m9.figshare.c.6904379) PMC10645078 37935356

